# Optimizing heart disease diagnosis with advanced machine learning models: a comparison of predictive performance

**DOI:** 10.1186/s12872-025-04627-6

**Published:** 2025-03-22

**Authors:** M Darshan Teja, G Mokesh Rayalu

**Affiliations:** Department of Mathematics, School of Advanced Sciences, Vellore Institute of Technology, Vellore, India

**Keywords:** Heart diseases, Machine learning models, Confusion matrix, ROC-AUC, Metrics

## Abstract

Cardiovascular disease is the leading cause of mortality globally, necessitating precise and prompt predictive instruments to enhance patient outcomes. In recent years, machine learning methodologies have demonstrated significant potential in enhancing the precision and efficacy of health-related predictions, especially in the identification of heart disease. The dataset used in this study came from the UC Irvine Machine Learning Repository and included data from Cleveland, Switzerland, Hungary, Long Beach, and Statlog. We selected seven of the 1,190 cases, each with 12 attributes, for analysis. We used different machine learning models, like Random Forest, K-Nearest Neighbors, Logistic Regression, Naïve Bayes, Gradient Boosting, AdaBoost, XGBoost, and Bagged Trees, to check performance using accuracy, precision, recall, F1-score, and ROC-AUC. K-fold cross-validation (K = 10, K = 5) was conducted to guarantee the robustness and generalizability of these models. Random Forest exhibited remarkable stability, attaining 94% accuracy with K = 10 and 92% with K = 5, whereas XGBoost had a minor decrease during cross-validation (90% for K = 10, 89% for K = 5). KNN demonstrated possible overfitting, evidenced by a notable decline in accuracy (71% for K = 10, 72% for K = 5). XGBoost and Bagged Trees achieved the highest accuracy of 93%, followed by Random Forest and KNN at 91%. Furthermore, Random Forest and Bagged Trees exhibited the highest ROC-AUC values at 95%, and XGBoost demonstrated a ROC-AUC of 94%. The results demonstrate the effectiveness of ensemble methods in predicting cardiac diseases, along with the potential for future advancement through the incorporation of hybrid models and advanced survival analysis techniques.

## Introduction

Cardiovascular disorders (CVDs) are the primary cause of mortality worldwide, resulting in 17.9 million deaths per year. Heart attacks and strokes are the leading causes, with one-third occurring prematurely in people under 70 [1]. Behavioral risk factors include unhealthy diet, inactivity, tobacco use, alcohol, and air pollution. Heart failure, stroke, and heart attacks are more likely in people with intermediate risk factors. In Cleveland, unhealthy diets, smoking, alcohol, fat, and tobacco use contribute to a 50–60% mortality rate [2]. In Hungary, cardiovascular diseases are among the most common causes of death, with 60,000 deaths annually [3]. In Switzerland, cardiovascular disease and cancer were the most common killers in 2022 [4]. Comprehensive datasets from Cleveland, Hungary, Switzerland, Long Beach, and Statlog demonstrate the global burden of cardiac disease and continuous attempts to study and treat these disorders through modern medical research and data analysis [5].

Machine learning (ML) is an effective method for analysing complicated information in a variety of industries, including healthcare. Machine learning (ML) approaches use training algorithms to find patterns and make predictions based on data without explicit programming [6]. A wide array of ML models, including Logistic Regression, Random Forest, Support Vector Machines, K-Nearest Neighbours, Gradient Boosting Machines, Neural Networks, XGBoost, Model Averaged Neural Networks, Flexible Discriminant Analysis, Conditional Inference Trees, Bagged Trees, Naive Bayes, Multivariate Adaptive Regression Splines, Boosted Generalised Linear Models, Random Trees, and Bayesian Generalised Linear Models, have been applied to various datasets to extract [7].

Machine learning techniques are critical for increasing the accuracy and efficiency of identifying cardiac disease. By analysing massive datasets, ML models can forecast the onset of cardiac disease, identify high-risk individuals, and recommend the best treatments. Performance criteria like as accuracy, recall, precision, and ROC-AUC are utilised to assess these models. High accuracy reflects the model’s capacity to correctly classify examples; recall evaluates the model’s ability to identify all relevant cases; precision evaluates the number of genuine positive findings; and ROC-AUC offers an overall measure of performance across all classification thresholds.

Through the application of cutting-edge machine learning methods, this research aims to provide a thorough examination of cardiac disease prognosis. The literature on heart disease prediction is reviewed in the Review of literature section, with an emphasis on earlier approaches and their results. The technique used for this study is described in the Methodology section, along with the steps involved in gathering data and the features that were selected. The dataset, together with its sources, features are all described in Data set. The measurements and descriptions of the machine learning models used are described in the section of Methods. The study and findings, which highlight the various models’ performances, are presented in Analysis and Results. In Discussion section, the results are compared with earlier studies, highlighting the improvements achieved in this investigation, makes recommendations for further development and finally we concludes in the last section. 

## Review of literature

Numerous academic contributions have investigated cardiac disease detection using data mining and machine learning approaches, drawing on datasets from Cleveland and other nations. Researchers have used a variety of methodologies, including ensemble learning, neural networks, logistic regression, and support vector machines, to improve prediction accuracy and dependability.

Chandrasekhar et al. (2023) employed machine learning to increase cardiovascular disease forecasting accuracy. They employed six approaches: random forest, K-nearest neighbour, logistic regression, Naïve Bayes, gradient boosting, and AdaBoost classifier. The Cleveland dataset produced the greatest results, with logistic regression reaching 90.16% accuracy. AdaBoost scored 90% accuracy on the IEEE Dataport dataset. A softly selected ensemble classifier integrating all six techniques improved performance to 93.44% and 95%, outperforming logistic regression and AdaBoost classifiers [8]. Perumal et al. (2020) They focus evaluated three classifiers: LR, SVM, and KNN, focusing on their performance metrics. LR and SVM outperformed the KNN classifier in terms of accuracy, sensitivity, specificity, and MCC. The LR classifier had higher sensitivity, specificity, accuracy, MCC, and error rate, while the SVM had lower performance [9].

Gao et al. (2021) created a system for predicting heart illness that employs ensemble approaches such as boosting and bagging, as well as PCA and Linear Discriminant Analysis are two examples of feature extraction methods. They utilised the Cleveland heart disease dataset to evaluate the performance of five classifiers. The bagging ensemble learning technique, when paired with Decision Trees and PCA feature extraction, produced the greatest results, with an amazing accuracy of 98.6%. This demonstrates the efficacy of integrating ensemble approaches with feature extraction algorithms for heart disease prediction [10]. kahramanli et al. (2008) The suggested medical categorization system was tested using the Pima Indian diabetes and Cleveland heart disease datasets. The performance measures employed were accuracy, sensitivity, and specificity. The technique obtained 84.24% and 86.8% accuracy for the heart disease datasets, respectively, which were among the best when compared to prior research and UCI websites [11].

Hassan D et al. (2023) suggest a novel technique for heart disease prediction based on a pre-trained DNN, PCA, and logistic regression. The Cleveland dataset had been used to assess the effectiveness of the suggested strategy. Experimental findings demonstrated that the suggested technique worked well on both training and testing data, with 91.79% and 93.33% accuracy rates, respectively [12]. Samuel et al. (2016) novel decision support system, which combines ANN and Fuzzy AHP approaches, was evaluated on 297 heart failure patients using an online clinical dataset. The system attained an average prediction accuracy of 91.10%, which is 4.40% greater than the standard ANN technique. It also outperformed seven earlier approaches, which ranged in accuracy from 57.85 to 89.01% [13].

Rahman et al. (2018) investigated the Cleveland heart disease dataset and used a variety of approaches to predict heart disease. They used Naive Bayes with a threshold of 0.5, resulting in an accuracy of 88.35%, and with a threshold of 0.4, they increased their accuracy to 89.32%. Logistic regression was also used, resulting in an accuracy of 84.47%. Furthermore, a Neural Network technique achieved a noteworthy accuracy of 90.2%. The suggested ensemble approach, RIHDPS, was the study’s most important contribution, with a maximum accuracy of 91.26%. This demonstrates how ensemble approaches improve forecast accuracy for heart disease [14].

For instance, B Shi et al. (2022) suggested a JASMA-SVM framework that uses clinical vitamin D and thyroid data to accurately predict recurrent spontaneous abortion. X Fei et al. (2020) added sparse learning to MEKLM to improve the use of neuroimaging to diagnose Alzheimer’s and Parkinson’s diseases. In the field of computer vision, L. Zang et al. (2021) created a deep active learning framework for scene classification that is based on biological inspiration. It uses object-based segmentation and multimodal feature fusion to get good results on remote sensing datasets. Additionally, Chen, M. R., Zeng, G. Q., & Lu, K. D. (2019) presented the MaOPEO-HM algorithm, a novel many-objective optimization approach with adaptive hybrid mutation, which outperformed several state-of-the-art evolutionary algorithms on benchmark problems [31].

**Table 1:** An overview of cutting-edge strategies for predicting cardiovascular illnesses. Individual classifiers

*R*. No	Author	Year	Data Set	Methods	Results (Accuracy)
[7]	Hassan et al.	2024	Cardiovascular diseases dataset	XGboost, Random Forest	85.23, 86.36
[15]	Almazroi et al.	2023	Cleveland, Hungarien, Switzerland, Long Beanch HDD	Deep Learning	83
[12]	Hassan D et al.,	2023	Cleveland HDD	DNN + PCA + LR	91.79–93.33
[9]	Perumal et al.;	2020	Coronary heart diseases from Cleveland	LR, SVM, KNN	85,87,69
[13]	Samuel et al.;	2016	Heart Failure Rate	ANN, Fuzzy AHP	91.10, 89.01
[10]	Gao et al.;	2021	Heart Diseases	PCA + DT	98.6
[14]	Rahman et al.;	2018	Cleveland heart diseases data set	Navie Bayes (0.4 and 0.5), LR, Neural Network, RIHDPS	89.32, 88.35, 84.47, 90.21, 91.26
[11]	Kahramanli et al.;	2008	Pima Indian Diabetes and Clever data set	ANN, Fuzzy Neural Network	84.24, 86.8
[8]	Chandrasekhar, N., & Peddakrishna, S.	2023	Clever HDD sets and IEEE Dataport data set	LR, AdaBoost	90.16,90
[16]	Singh, A., & Kumar, R.	2020	UCI repository dataset	SVM, DT, Linear Regression	83, 79, 78
[17]	Maji, S., & Arora, S.	2019	UCI respository	DT, ANN	78,77
[18]	Senthilkumar et al.	2019	UCI respository heart diseases dataset	HRLM	88.7
[19]	Amin et al.	2019	Heart diseases data set	VOTE( NB + LR)	87.4
[20]	Chun-an cheng & Hung-wen chiu	2017	UCI laboratory heart diseases data	Hybrid model for NN, DT, SVM, Naïve bayes	86.8
[21]	Reddy, G. T., & Khare, N.	2017	Cleveland, Hungarian and Switzerland datasets	Radial Basis Network Link Network	78
[22]	Khateeb, N., & Usman, M.	2017	HDD	KNN	80

Table 1 summarizes the work of many authors on heart disease data, including research that used the same dataset, as well as their findings. However, in our study, we focused on a subset of features are sex, chest pain type, fasting blood sugar, resting ECG, exercise angina, ST slope, and target. Unlike prior research, which failed to find ROC-AUC, our study contains this critical statistic. By adding ROC-AUC, we can provide a more thorough evaluation of the model’s performance, filling a gap in the literature and establishing a more rigorous framework for heart disease prediction. This inclusion of ROC-AUC guarantees that the models’ diagnostic capacity is properly evaluated, increasing the dependability of the results.

## Methodology

**Fig. 1:**
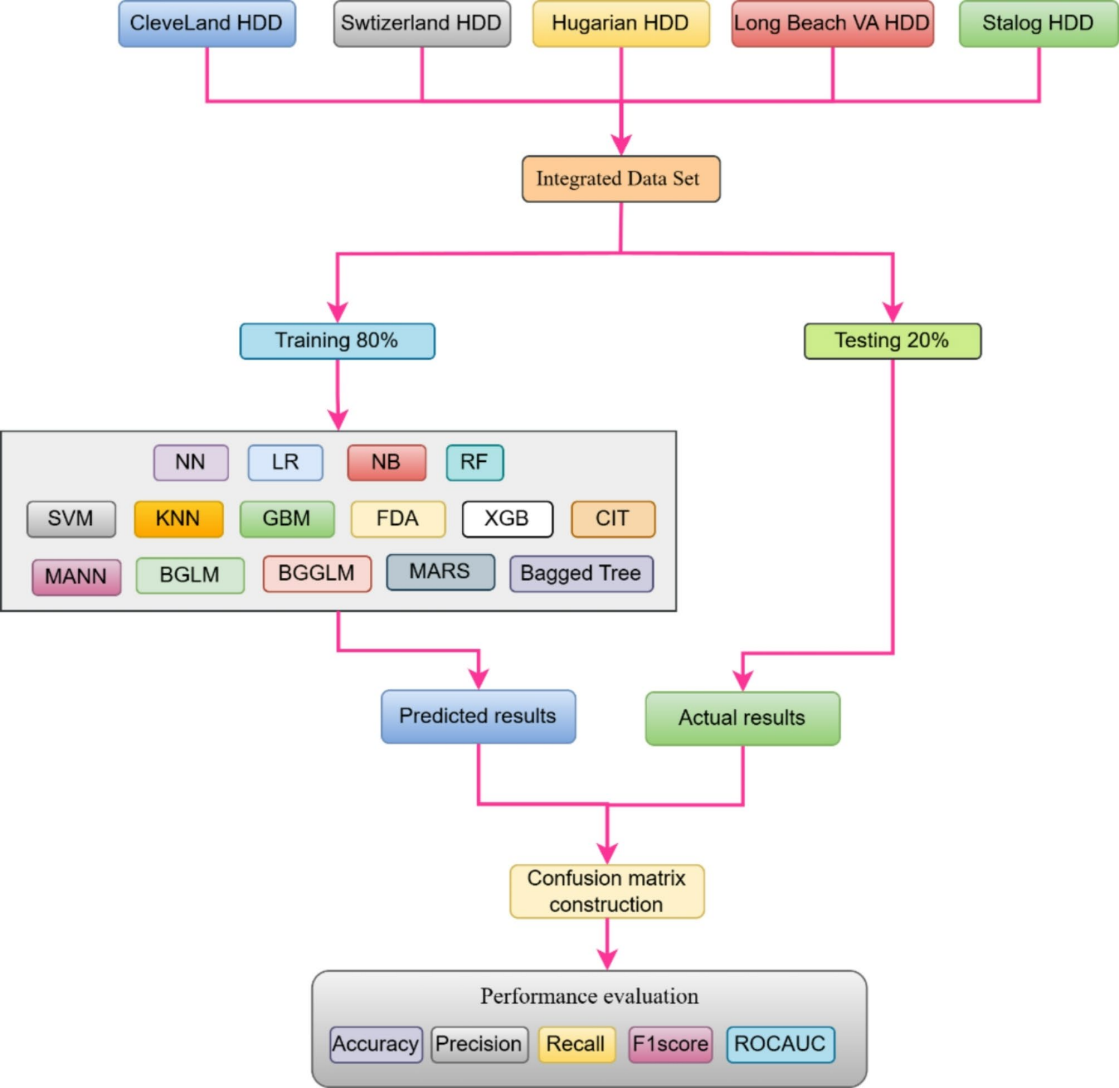
Proposal methodology for analysis

This study used data from the UCI Repository, which included five separate heart disease datasets: Cleveland, Switzerland, Hungarian, Long Beach VA, and Statlog. The merged dataset comprised of 1190 records and 12 characteristics. For the purpose of this study, only seven relevant characteristics were chosen for investigation. The dataset was divided into training and testing sets with an 8:2 ratio. Following the data splitting, 15 machine learning models were applied to the dataset. The performance of these models was measured using a variety of measures, including accuracy, precision, F1 score, recall, confusion matrix, and ROC-AUC. This extensive examination enabled a detailed comparison of the models’ performance in predicting cardiac disease as overall process is shown in the Fig. 1.

## Data set

The heart disease dataset utilised in this work was acquired from the UC Irvine Machine Learning Repository and Kaggle, and it included 12 features and 1,190 entries. The collection is made up of data from several sites, including Cleveland (303 records), Hungary (294 recordings), Switzerland (123 records), Long Beach, Va (200 records), and Stalog (270 records). Combining all of these datasets yielded a comprehensive dataset of 1,190 records. For our research, we chose the seven most important variables from the original 12, with an emphasis on those that make a major contribution to heart disease prediction. This meticulous selection is intended to improve the performance and accuracy of the various machine learning models used in this study.

**Table 2:** Data set description

S.No	Variable Name	Description and range	Feature Type
1.	Age	Age 30 to 90	Integer
2.	Sex	0-female, 1-male	Binary
3.	Chest-pain	Chest pain type 1 to 4	Nominal
4.	Rest-bp	Resting Blood Pressure 90 to 180	Integer
5.	Serum-chol	Serum Cholestrol mg/dl [126,564]	Integer
6.	Fastingbloodsugar (FBS)	FBS above 120 mg/dl [1 = true, 0 = false]	Binary
7.	Electrocardiographic (ECG)	Resting electrocardiographic results	Nominal
8.	Max-heart-rate	Maximum heart rate achieved 90 to 180	Integer
9.	Angina	exercise induced angina [0 = no, 1 = yes]	Binary
10.	Old peak	oldpeak = ST depression induced by exercise relative to rest	Integer
11.	Slope	the slope of the peak exercise ST segment	Normal
12.	Target	0-No, 1-Yes	Binary

We selected seven key variables for analysis from Table 2, which contained dataset details: sex, chest pain kind, fasting blood sugar, resting ECG, exercise angina, ST slope, and Target. These characteristics were chosen based on their relation to heart disease prognosis. Using these chosen characteristics, multiple machine learning algorithms were applied to the dataset to predict heart disease outcomes. This concentrated strategy is intended to improve the predicted accuracy and reliability of the models by focusing on the most useful factors.

## Methods

### Metrics

**Confusion matrix**:

A confusion matrix is a tabular format that evaluates the effectiveness of a classification model. It juxtaposes the anticipated classifications with the actual classifications, yielding insights about the model’s performance efficacy. The matrix comprises four fundamental elements: True Positives (TP), True Negatives (TN), False Positives (FP), and False Negatives (FN).

$$\:CM=\left[\begin{array}{cc}TP&\:FN\\\:FP&\:TN\end{array}\right]$$

**Accuracy**:

Accuracy is the fraction of accurately predicted cases (including true positives and true negatives) among all instances.

1 $$\:Accuracy=\frac{TP+TN}{TP+TN+FP+FN}$$

**Precision**:

Precision is also known as positivity. The predictive value is the proportion of real positive forecasts among all positive ones.

2 $$\:Precision=\:\frac{TP}{TP+FP}$$

**Recall**:

Recall, also known as responsiveness or true positive rate, is a metric that indicates the proportion of real positive forecasts across all positives.

3 $$\:Recall=\:\frac{TP}{TP+FN}$$

**F1Score**:

The score of F1 is the harmonic average of precision and recall, resulting in a balance of the two measures [23].

4 $$\:F1\:score=2\:X\frac{Precision\:X\:Recall}{Precision+Recall}$$

**ROC-AUC (Receiver Operating Characteristic– Area Under the curve)**

The Recall (TPR) and False Positive Rate (FPR) are contrasted on the ROC curve. The classifier’s performance is summarised at every level of classification by the AUC (Area Under the Curve) [24].

5 $$\:AUC=\underset{0}{\overset{1}{\int\:}}TPRd\left(FPR\right)$$

### Models

**Logistic regression**:

A statistical model called logistic regression uses binary classification to forecast if an input is part of a particular class or not. The logistic function is employed to translate the output into probability values [20].

6 $$\begin{array}{l}\\\:P\left(Y=1|X\right)=\\\:\frac{1}{1+{e}^{-({\beta\:}_{0}+{\beta\:}_{1}{X}_{1}+\dots\:\dots\:\dots\:\dots\:.+{\beta\:}_{n}{X}_{n})}}\end{array}$$

**Random forest**:

As part of a group learning technique called random forest, many decision trees are built during training and the mean estimate for regression of each tree or the mode of the categories for classification are returned.

7 $$\:\widehat{f}\left(x\right)=\frac{1}{B}\sum\:_{b=1}^{B}{f}_{b}\left(x\right)$$

Where $\:\widehat{f}\left(x\right)$ is the aggregated prediction from B trees [22].

**SVR (Support Vector Regression)**.

SVR is a support vector machine that solves regression issues. It seeks a function that deviates by no more than e from the actual goal values.

8 $$\:f\left(x\right)=\:\sum\:_{i=1}^{n}\left({\alpha\:}_{i}-{{\alpha\:}_{i}}^{*}\right)K\left({x}_{i},x\right)+b\:$$

Where $\:K\left({x}_{i},x\right)$ is the Kernel function.

**KNN (K-nearest neighbors)**

KNN is a slow, non-parametric learning method that may be applied to regression and classification. The average of the values of the KNN is the result of regression.

9 $$\:\widehat{f}\left(x\right)=\frac{1}{k}\sum\:_{i\in\:{N}_{k}\left(x\right)}{y}_{i}$$

Where $\:{N}_{k}\left(x\right)$ are the k-nearest neighbors of x.

**GBM (gradient boosting machine)**

Gradient Boosting Machine is an ensemble learning approach that constructs a model stage by stage using weak learners such as decision trees and optimises a loss function using gradient descent.

10 $$\:{F}_{m}\left(x\right)={F}_{m-1}\left(x\right)+v{h}_{m}\left(x\right)$$

Where $\:{h}_{m}\left(x\right)$ is the base learner, $\:v$ is the learning rate.

**Neural network**:

A neural network is a set of algorithms that attempt to recognise correlations in data using an approach comparable to the human brain. It consists of layers of nodes, or neurons, that are linked in a network [23, 26].

11 $$\:{a}^{\left(l\right)}=g\left({W}^{\left(l\right)}{a}^{\left(l-1\right)}\right)+{b}^{\left(l\right)}$$

Where g is the activation function, $\:{W}^{\left(l\right)}$ and $\:{b}^{\left(l\right)}\:$are the weights and biases of layer l.

**XGBoost**

XGBoost (Extreme Gradient Boosting) is an optimised gradient boosting system that use decision trees and is intended for speed and performance.

12 $$\:{F}_{m}\left(x\right)={F}_{m-1}\left(x\right)+{\gamma\:}_{m}{h}_{m}\left(x\right)$$

Where $\:{\gamma\:}_{m}$ is the weight of the mth tree, and $\:{h}_{m}\left(x\right)$ is the m-th tree’s prediction.

**Model averaged neural network (MANN)**

To increase generalisation and prevent overfitting, model-averaged neural networks are trained using several neural networks and then averaged to produce predictions. This ensemble technique combines the strengths of individual models [21].

13 $$\:\widehat{y}=\frac{1}{M}\sum\:_{m=1}^{M}{\widehat{y}}^{\left(m\right)}$$

**Flexible discriminant analysis (FDA)**

Flexible discriminant analysis extends linear discriminant analysis by including non-linear correlations through the use of basis expansions and non-parametric regression strategies.

14 $$\:{g}_{k}\left(x\right)=\sum\:_{j=1}^{p}{\varnothing\:}_{j}\left(x\right){\beta\:}_{jk}$$

Where $\:{\varnothing\:}_{j}\left(x\right)$ are basis functions and $\:{\beta\:}_{jk}$ are coefficients for class k.

**Conditional inference tree (CIT)**

Conditional inference trees are a sort of decision tree that selects splits using hypothesis testing, therefore avoiding the overfitting and selection bias found in regular decision trees.

The splitting criterion is based on the test statistic:

15 $${\rm{Test}}\,{\rm{statistic = }}\:ma{x_j}\left( {\frac{{\left| {{T_j}} \right|}}{{\sigma {\:_j}}}} \right)\:$$

Where $\:{T}_{j}$ is the test statistic for predictor j and $\:{\sigma\:}_{j}$ is its standard error.

**Bagged tree (BT)**

Bagged Trees are an ensemble approach that creates numerous decision trees using distinct bootstrap samples of training data and then aggregates their results. This helps to minimise variation and increase model stability.

16 $$\:\widehat{{f}_{b}}\left(x\right)=\frac{1}{T}\sum\:_{t=1}^{T}{f}_{t}\left(x\right)\:$$

Where T is the number of bootstrap samples and $\:{f}_{t}\left(x\right)$ is the prediction from the t-th tree.

**Navie bayes (NB)**

Navie bayes is a probabilistic classifier based on the Bayes theorem, which assumes predictor independence. It determines the posterior probability of each class based on the supplied characteristics [25].

17 $$\:P\left({C}_{k}|x\right)=\:\frac{P\left({C}_{k}\right)\prod\:_{i=1}^{n}P\left({x}_{i}\right|{C}_{k})}{P\left(x\right)}$$

Where $\:{C}_{k}$is the class, $\:{x}_{i}$ are the input features, and P(x) is the marginal probability of x.

**Multivariate adaptive regression splines (MARS)**

MARS is a regression approach that models relationships by fitting piecewise linear regressions known as splines, which automatically pick and combine predictor variables to increase prediction accuracy.

18 $$\:f\left(x\right)={\beta\:}_{0}+\sum\:_{m=1}^{M}{\beta\:}_{m}{h}_{m}\left(x\right)$$

Where $\:{h}_{m}\left(x\right)$ are basis functions and $\:{\beta\:}_{m}\:$ are coefficients.

**Boosted generalized linear model (BGGLM)**

Boosted The generalised linear model combines the flexibility of boosting methods with the interpretability of generalised linear models, iteratively refining the model through residual fitting [27].

19 $$\:{F}_{m}\left(x\right)={F}_{m-1}\left(x\right)+v.{GLM}_{m}\left(x\right)$$

Where v is the learning rate, and $\:{GLM}_{m}\left(x\right)$ is the m-th GLM fit to the residuals.

**Bayesian generalized linear model (BGLM)**

The Bayesian Generalised Linear Model (BGLM) builds on generalised linear models by including Bayesian inference, which allows for the integration of previous information and probabilistic interpretations of model parameters.

20 $$\:p\left(\beta\:\right|y)\propto\:p\left(y|\beta\:\right)p(\beta\:)$$

Where $\:p\left(y|\beta\:\right)$ is the likelihood and $\:p\left(\beta\:\right)$ is the prior distribution.

## Analysis and results

### Feature selection

**Fig. 2:**
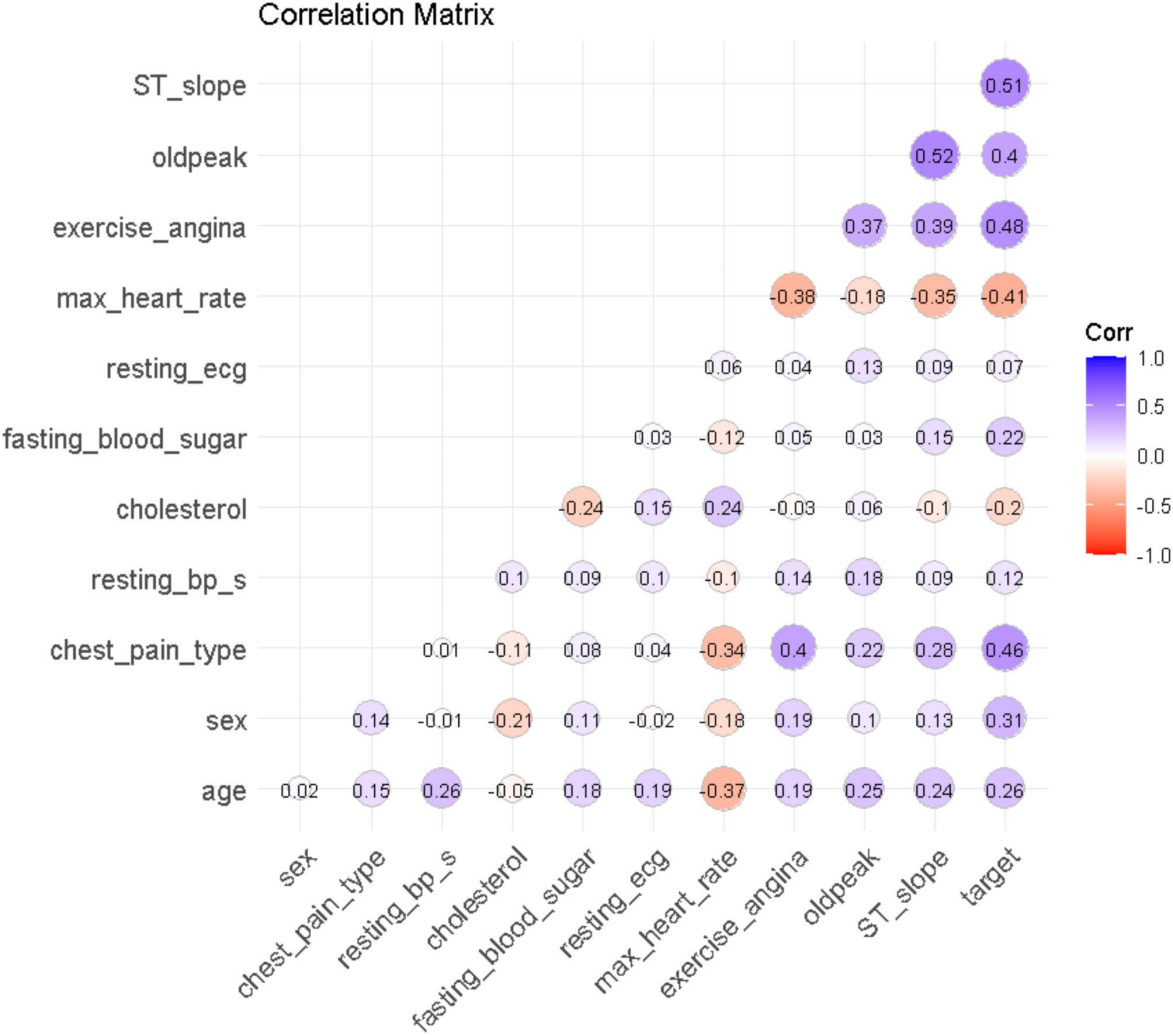
The findings of correlation across all characteristics are shown as a heatmap

The heart disease dataset’s correlation matrix reveals significant relationships between various variables in Fig. 2. Age is moderately correlated with resting blood pressure and exercise-induced angina, suggesting older individuals may have higher blood pressure and angina during exercise. Sex is positively correlated with the target variable, indicating a higher likelihood of heart disease in males. Chest pain type is strongly correlated with exercise-induced angina and the target variable, underscoring its importance in heart disease diagnosis. Max heart rate has a significant negative correlation with age and the target variable, suggesting higher heart rates are associated with younger age and lower heart disease prevalence. Exercise-induced angina and ST slope have strong positive correlations with the target variable, and old peak is positively correlated with ST slope and the target variable.

Many research studies have shown that different kinds of chest pain, like angina, atypical angina, and non-anginal pain, are strong signs that someone might get heart disease. Researchers have derived their findings from these studies [32, 33]. The Framingham Heart Study came to the conclusion that elevated levels of blood sugar in the fasting state are associated with an increased risk of cardiovascular diseases [34, 35]. This was one of the findings of the study. An important diagnostic tool for figuring out if someone has had a myocardial infarction or ischemia is finding any changes in the data from an ECG [36]. In the presence of ischemic heart disease, there is a strong association between the occurrence of exercise-induced angina and the risk of developing the condition. When attempting to diagnose coronary artery disease, the slope of the ST segment at maximal activity is a highly essential component to consider [37]. We chose seven features or variables to use machine learning analysis on based on the results of correlation and another research paper.

### Evaluation metrics for machine learning models

In this work, we want to predict heart disease by analyzing a dataset that originally included 12 Features or variables. To enhance the functionality of the model, we carefully chose the seven most relevant variables. Applying these variables to different machine learning models produces substantial outcomes. Logistic regression has an accuracy of 0.83, a precision of 0.84, a recall of 0.78, and F1 score of 0.81. Random Forest performs admirably, with an accuracy of 0.91, a precision of 0.92, a recall of 0.90, and an F1 score of 0.91. SVM regression produces comparable results as logistic regression, whereas KNN matches Random Forest’s performance with an accuracy of 0.91. GBM has an accuracy of 0.87, with balanced precision and recall scores. Neural networks and model-averaged neural networks both score an accuracy of 0.85.

XGBoost has the best accuracy of 0.93, precision of 0.92, recall of 0.92, and F1 score of 0.92. Flexible Discriminant Analysis (FDA) and MARS both have an accuracy of 0.84, with minor differences in precision and recall. The Conditional Inference Tree (CIT) model has a lower accuracy of 0.81, while other metrics are marginally lower than those of the FDA and MARS. The Bagged Tree model is comparable to XGBoost’s strong performance, with an accuracy of 0.93 and continuously good precision and recall. Naive Bayes obtains an acceptable accuracy of 0.85.

Bayesian Generalised Linear Models (BGLM) and Boosted Generalised Linear Models (BGGLM) perform similarly, with accuracies of 0.83 and 0.835, respectively as results shown in Table 3. This investigation compares the efficacy of several machine learning models in predicting cardiac disease, demonstrating the value of choosing essential factors to improve model performance.

**Table 3:** Individual classifier results were evaluated

S.No	Models	Accuracy	Precision	Recall	F1Score
1	Logistic Regression	0.83	0.84	0.78	0.81
2	Random Forest	0.91	0.92	0.90	0.91
3	SVM Regression	0.83	0.84	0.79	0.82
4	KNN	0.91	0.91	0.91	0.91
5	GBM	0.87	0.87	0.86	0.86
6	Neural Network	0.84	0.85	0.80	0.82
7	XGBoost	0.93	0.92	0.92	0.92
8	MANN	0.84	0.86	0.79	0.82
9	FDA	0.84	0.85	0.82	0.83
10	CIT	0.81	0.78	0.82	0.80
11	Bagged tree	0.93	0.93	0.92	0.93
12	Navi Bayes	0.85	0.87	0.80	0.83
13	MARS	0.84	0.85	0.82	0.83
14	BGLM	0.83	0.84	0.80	0.82
15	BGGLM	0.83	0.84	0.79	0.82

### ROC-AUC

**Fig. 3:**
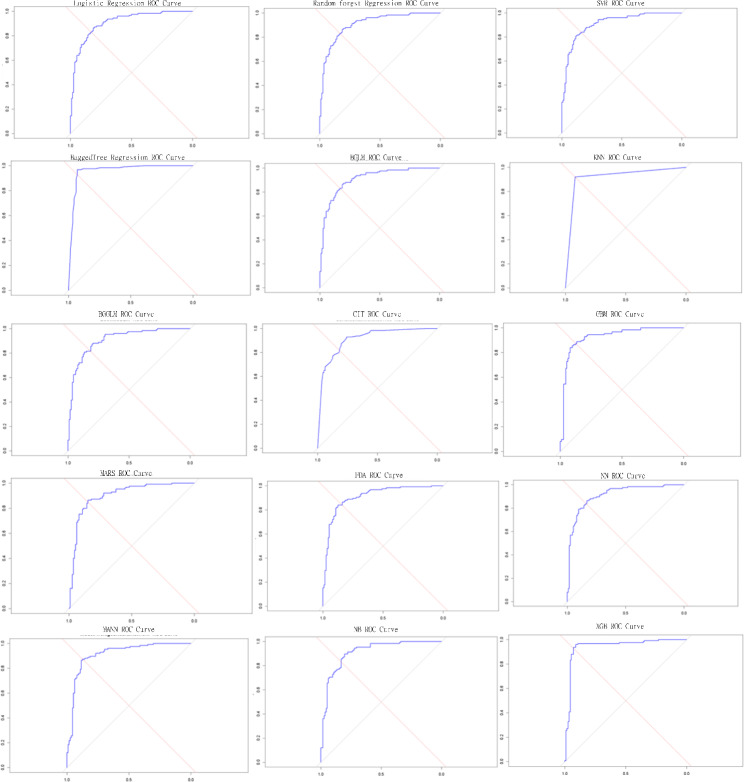
ROC-AUC plot for different Machine learning models

The comparison of multiple machine learning models on the heart disease dataset using ROC-AUC (Receiver Operating Characteristic-Area Under the Curve) metrics indicates considerable disparities in predicting ability mention in Table 4; Fig. 3 (where x-axis represents Specificity and y-axis represents Sensitivity). Notably, ensemble approaches such as Random Forest and Bagged Tree had the highest ROC-AUC values of 0.95, showing a higher capacity to identify between individuals with and without heart disease. XGBoost also scored admirably, with a ROC-AUC of 0.94, indicating its capability to handle complicated data structures and capture intricate patterns within the dataset. The Gradient Boosting Machine (GBM) had a ROC-AUC of 0.92, whereas models such as SVM regression, KNN, Neural Network, MANN, FDA, CIT, and Naïve Bayes consistently had ROC-AUC values of 0.91, showing their dependability in prediction tasks.

Traditional models, such as Logistic Regression, MARS, BGLM, and BGGLM, performed marginally worse, with ROC-AUC values of 0.90. While these models still have decent accuracy, the results show that ensemble methods and advanced boosting techniques outperform them in terms of predictive power. These findings highlight the significance of model selection and optimisation in heart disease prediction, implying that combining powerful ensemble algorithms might greatly improve diagnostic accuracy. Furthermore, the higher performance of models such as Random Forest and Bagged Tree, together with the promising results of XGBoost, suggests that utilising ensemble learning approaches may lead to more reliable and accurate cardiac disease prediction systems, ultimately improving patient outcomes.

**Table 4:** ROC-AUC values for machine learning models

S.No	Models	AUC
1.	Logistic Regression	0.90
2.	Random Forest	0.95
3.	SVM regression	0.91
4.	KNN	0.91
5.	GBM	0.92
6.	Neural Network	0.91
7.	XGBoost	0.94
8.	MANN	0.91
9.	FDA	0.91
10.	CIT	0.91
11.	Bagged Tree	0.95
12.	Navie Bayes	0.91
13.	MARS	0.90
14.	BGLM	0.90
15.	BGGLM	0.90

### Confusion matrix

**Table 5:** Confusion matrix for different models

S.No	Models	Confusion Matrix
1	Logistic Regression	$$\:\left[\begin{array}{cc}88&\:16\\\:24&\:109\end{array}\right]$$
2	Random forest	$$\:\left[\begin{array}{cc}101&\:8\\\:11&\:117\end{array}\right]$$
3	SVM Regression	$$\:\left[\begin{array}{cc}89&\:16\\\:23&\:109\end{array}\right]$$
4	KNN	$$\:\left[\begin{array}{cc}103&\:10\\\:9&\:115\end{array}\right]$$
5	GBM	$$\:\left[\begin{array}{cc}97&\:14\\\:15&\:111\end{array}\right]$$
6	Neural Network	$$\:\left[\begin{array}{cc}90&\:15\\\:12&\:110\end{array}\right]$$
7	XGBoost	$$\:\left[\begin{array}{cc}104&\:8\\\:8&\:117\end{array}\right]$$
8	MANN	$$\:\left[\begin{array}{cc}89&\:14\\\:23&\:111\end{array}\right]$$
9	FDA	$$\:\left[\begin{array}{cc}92&\:16\\\:20&\:109\end{array}\right]$$
10	CIT	$$\:\left[\begin{array}{cc}92&\:25\\\:20&\:100\end{array}\right]$$
11	Bagged Tree	$$\:\left[\begin{array}{cc}104&\:7\\\:8&\:118\end{array}\right]$$
12	Navi Bayes	$$\:\left[\begin{array}{cc}90&\:11\\\:22&\:112\end{array}\right]$$
13	MARS	$$\:\left[\begin{array}{cc}92&\:16\\\:20&\:109\end{array}\right]$$
14	BGLM	$$\:\left[\begin{array}{cc}90&\:17\\\:22&\:108\end{array}\right]$$
15	BGGLM	$$\:\left[\begin{array}{cc}89&\:16\\\:23&\:109\end{array}\right]$$

Overall, Random Forest, XGBoost, and Bagged Tree models outperform in predicting heart disease, with the lowest misclassification rates and best accuracy in confusion matrix. KNN, GBM, and Neural Networks all perform well, making them reliable prediction models. Models such as Logistic Regression, SVM, FDA, MANN, and Naive Bayes perform well but with greater error rates, whereas CIT, BGLM, and BGGLM have lesser accuracy and may require more optimisation for better performance from Table 5.

### Cross-Validation

**Table 6:** K-fold Cross-Validation accuracy for machine learning models

S.No	Models	Accuracy	K = 10 Accuracy	K = 5 Accuracy
1	Logistic Regression	0.83	0.84	0.84
2	Random Forest	0.91	0.94	0.92
3	SVM Regression	0.83	0.86	0.85
4	KNN	0.91	0.71	0.72
5	GBM	0.87	0.89	0.86
6	Neural Network	0.84	0.85	0.84
7	XGBoost	0.93	0.90	0.89
8	MANN	0.84	0.85	0.84
9	FDA	0.84	0.85	0.84
10	CIT	0.81	0.83	0.82
11	Bagged tree	0.93	0.91	0.92
12	Navi Bayes	0.85	0.84	0.83
13	MARS	0.84	0.85	0.84
14	BGLM	0.83	0.84	0.83
15	BGGLM	0.83	0.84	0.84

This study uses a combined dataset from several sources to show how well different machine learning models predict cardiac disease. We significantly improved predicted performance by applying fifteen different models and concentrating on seven key features. The maximum accuracy was 93% for XGBoost and Bagged Trees and 91% for Random Forest and KNN. Cross-validation results showed that XGBoost performance slightly decreased while Random Forest performance stayed high (94% for K = 10 and 92% for K = 5). This added to the idea that the model was stable. ROC-AUC values showed that Random Forest and Bagged Trees got 95%, XGBoost got 94%, and GBM got 92%. This showed that the models were more reliable. Notably, under cross-validation, KNN’s accuracy dramatically decreased, suggesting possible overfitting. Conventional models like Naïve Bayes, SVM Regression, and Logistic Regression performed moderately yet consistently. The results demonstrate in the Table 6, to evaluate models for more than just accuracy because cross-validation offers a reliable assessment of their ability to generalize. Taking everything into account, our results surpass those of previous studies, showcasing the potential of advanced machine learning techniques for precise clinical cardiac disease forecasting.

## Discussion

Table 1 summarizes the work of many writers on heart disease datasets, describing different approaches and their accuracies. Almazraoj et al. [15] achieved 83% accuracy with deep learning, whereas Singh et al. achieved 83% accuracy with SVM, 79% with decision trees, and 78% with linear regression. Maji et al. [17] discovered that decision trees and artificial neural networks (ANN) had accuracies of 78% and 77%, respectively, whereas Reddy et al. [21] reached 78% using RBNN. In our investigation, we employed a subset of characteristics and sophisticated machine learning algorithms to get much improved accuracy. XGBoost and Bagged Trees obtained 93% accuracy, while Random Forest and KNN got 91%. In addition, we computed ROC-AUC values, which were not addressed in earlier investigations. Our findings suggest that Random Forest and Bagged Trees have ROC-AUCs of 95, XGBoost has 94, GBM has 92, and the remaining models vary between 90 and 91, as shown in Table 4. This extensive examination indicates the higher performance and diagnostic capacity of our chosen models when compared to earlier efforts.

### Limitations

- Lack of Comprehensive Feature Evaluation: Many earlier studies used all accessible characteristics without rigorously examining their impact on model performance. Our study identifies seven significant variables based on domain expertise and assesses their influence with machine learning models.
- Limited Comparison of Machine Learning Models: Before we looked at ensemble methods, most study only looked at a few models, like logistic regression, decision trees, or neural networks. To find the best method, our study compares 15 models, such as advanced ensemble methods like XGBoost and Bagged trees.
- Absence of ROC-AUC Analysis: Some studies said they were accurate, but they didn’t give ROC-AUC scores, which are a more accurate way to measure how well a classification system works. This study fills in the blanks by looking at the ROC-AUC values of all the models, which gives a strong assessment of how well they can predict.
- Lack of Model Validation Techniques: A number of earlier studies may have overfitted because they failed to use appropriate validation procedures. K-fold cross-validation is used in our study to guarantee accurate and broadly applicable findings.
- Limited Model Interpretability and Deployment consideration: Some studies didn’t look at how the results would work in the real world, like how to make the computer work faster or how to make it easier to use models. Our work talks about the trade-offs between model complexity and performance, which can be used in real life.

### Future directions

- Exploring Survival Analysis Models: In the future, researchers could look into Gaussian Process Regression and other methods for survival analysis to figure out how heart diseases will get worse over time.
- Hybrid Model Approaches: It’s possible that combining several learning models (like XGBoost, Random Forest, and Bagged Trees) through ensemble or meta-analysis methods could make predictions more accurate overall.
- Real-time clinical Application: Incorporating the created models into a clinical decision support system could assist clinicians in the early diagnosis of cardiac disease and enhance patient outcomes.
- Validation on larger, Real-world Datasets: In the future, bigger, real-world datasets from hospitals or electronic health records (EHRs) should be used to test the model’s generalizability and practical applicability.

## Conclusion

This work offers a thorough assessment of machine learning models for predicting cardiac disease, utilizing a consolidated dataset from many sources. We employed fifteen distinct models, including XGBoost, Random Forest, Bagged Trees, and conventional classifiers such as Logistic Regression and Naïve Bayes, by picking seven essential features. XGBoost and Bagged Trees attained the highest accuracy of 93%, closely followed by Random Forest and KNN at 91%. To guarantee model robustness, we utilized k-fold cross-validation (K = 10, K = 5), wherein Random Forest showed enhanced stability (94% for K = 10, 92% for K = 5), while XGBoost displayed a marginal decrease. The ROC-AUC scores showed that the models were even more reliable. Random Forest and Bagged Trees got 95%, XGBoost got 94%, and GBM got 92%. Furthermore, tests such as precision, recall, and F1-score proved that these models worked, with Bagged Trees and Random Forest showing a strong balance between sensitivity and specificity. The confusion matrix elucidated misclassification tendencies, facilitating model enhancement. KNN demonstrated a propensity for overfitting, as its accuracy markedly declined during cross-validation. These results show that ensemble models are better at predicting heart disease and stress how important cross-validation is for generalizability. Our method is better than what has been done before, which shows that modern machine learning techniques can help doctors make accurate and reliable decisions.

## Data Availability

The data is secondary, obtained from the UCI Machine Learning Repository. https://archive.ics.uci.edu/.
